# Exploiting Electrostatic Interaction for Highly Sensitive Detection of Tumor-Derived Extracellular Vesicles by an Electrokinetic Sensor

**DOI:** 10.1021/acsami.1c13192

**Published:** 2021-09-02

**Authors:** Siddharth Sourabh Sahu, Sara Cavallaro, Petra Hååg, Ábel Nagy, Amelie Eriksson Karlström, Rolf Lewensohn, Kristina Viktorsson, Jan Linnros, Apurba Dev

**Affiliations:** † Department of Electrical Engineering, The Ångström Laboratory, Uppsala University, 75121 Uppsala, Sweden; ‡ Department of Applied Physics, School of Engineering Sciences, KTH Royal Institute of Technology, 10691 Stockholm, Sweden; § Department of Oncology-Pathology, Karolinska Institutet, 17164 Stockholm, Sweden; ∥ Department of Protein Science, School of Chemistry, Biotechnology, and Health (CBH), KTH Royal Institute of Technology, 10691 Stockholm, Sweden; ⊥ Theme Cancer, Patient Area Head and Neck, Lung, and Skin, Karolinska University Hospital, 17164 Solna, Sweden

**Keywords:** streaming current, electrokinetic method, charge modulation, enhanced sensitivity, extracellular vesicles, surface proteins, lung cancer, treatment monitoring

## Abstract

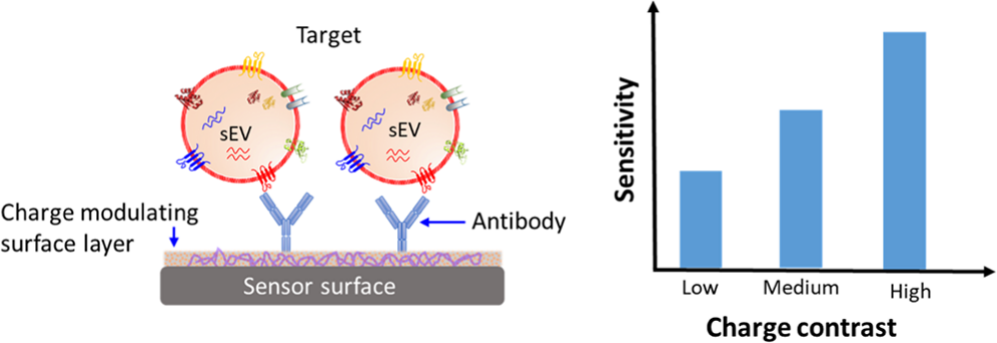

We present an approach to improve the detection sensitivity of a streaming current-based biosensor for membrane protein profiling of small extracellular vesicles (sEVs). The experimental approach, supported by theoretical investigation, exploits electrostatic charge contrast between the sensor surface and target analytes to enhance the detection sensitivity. We first demonstrate the feasibility of the approach using different chemical functionalization schemes to modulate the zeta potential of the sensor surface in a range −16.0 to −32.8 mV. Thereafter, we examine the sensitivity of the sensor surface across this range of zeta potential to determine the optimal functionalization scheme. The limit of detection (LOD) varied by 2 orders of magnitude across this range, reaching a value of 4.9 × 10^6^ particles/mL for the best performing surface for CD9. We then used the optimized surface to profile CD9, EGFR, and PD-L1 surface proteins of sEVs derived from non-small cell lung cancer (NSCLC) cell-line H1975, before and after treatment with EGFR tyrosine kinase inhibitors, as well as sEVs derived from pleural effusion fluid of NSCLC adenocarcinoma patients. Our results show the feasibility to monitor CD9, EGFR, and PD-L1 expression on the sEV surface, illustrating a good prospect of the method for clinical application.

## Introduction

Surface-based biosensors have received a lot of interest for developing highly sensitive, multiplexed, and lab-on chip compatible biosensors.^1−3^ A primary design consideration when developing such a sensor is its sensitivity, which has consequently motivated intense research interest. Since the response of such a sensor is proportional to the surface coverage of the analyte, a common strategy for enhanced sensitivity has been increasing the surface concentration of analytes by improving their mass transport rate.^4,5^ Another strategy has been to exploit the nature of the sensing principle to amplify the signal transduction, e.g., surface engineering,^6,7^ electrode design,^8,9^ and so on. In this context, the chemical surface functionalization, used in most affinity-based biosensors, may be exploited for an enhanced sensitivity. Indeed, over the years, a large variety of surface functionalization approaches have been developed.^10−12^ In most methodologies, the choice of surface functionalization approach is mainly motivated by its simplicity, compatibility with the sensing method, and the ability to generate higher probe density. However, given that such chemical functionalization drastically changes the physical and electrical properties of the interface layer, their influence on the sensor performance requires systematic investigation.

In our previous studies, we have shown that the sensitivity of electrokinetic sensor utilizing streaming current-based method strongly depends on the isoelectric point (pI) of the target.^13,14^ The studies were performed using analytes having different pI while keeping the electrostatic property of the surface similar. In fact, several theoretical and experimental studies have been performed to understand how different surface properties, e.g., the structural and electrical parameters of the surface, influence the sensitivity of such devices.^15−17^ These studies have indicated opportunities to further improve the detection sensitivity. For example, a large charge contrast between the sensor surface and the target is expected to result in a better sensitivity. In this case, surface functionalization used for immobilizing affinity probes, e.g., antibodies, may provide a suitable means to achieve a high charge contrast for a given target. For instance, for the detection of target molecules having a low isoelectric point, i.e., with a pI lower than the pH of the buffer, the surface can be modified by anchoring positively charged molecules and vice versa. A variety of polymer-based materials such as poly-l-lysine (PLL),^18,19^ poly(pentafluorophenyl acrylate,^20^ etc., are commercially available and can be easily anchored to a variety of substrates. The polymeric backbone can be further modified with various functional groups bearing different isoelectric points, thereby tuning the surface charge for an improved sensitivity. To the best of our knowledge, such a strategy has never been explored for improving the sensitivity of streaming current-based detection approach. The study can also guide the development of other surface-based sensors that rely on electrostatics for signal transduction.

In this study, we investigated the influence of charge contrast as a strategy to enhance the detection sensitivity of sEVs’ surface proteins. sEVs are a heterogeneous group of lipid-bilayer nanovesicles released by all cell types. They have recently attracted considerable research interest as potential sources of biomarkers for a large number of diseases, including cancer.^21^ We previously demonstrated the prospect of using a streaming current-based technique for profiling sEV-membrane proteins.^22^ These analyses were done by a standard functionalization strategy that included a chemical crosslinking reaction, mediated by glutaraldehyde, between a silanized silica surface^22^ and the primary amines of the affinity reagents. This method however does not cause sufficient electric-field screening of the underlying negatively charged silica surface; hence, the functionalized surface still remains highly negative. Considering that sEVs are also negatively charged,^23^ there is an obvious rationale to investigate if the charge contrast between a functionalized surface and sEVs can be further enhanced. To study this aspect, we evaluated three different functionalization schemes for antibody immobilization and determined the strategy that optimizes the charge contrast. An improvement in the limit of detection (LOD) by 2 orders of magnitude was recorded for the optimal functionalization strategy, which was in good agreement with the simulation performed using an existing theoretical model. The optimized sensing method was then applied to study the prospect of using sEVs for monitoring the efficacy of targeted cancer treatments. Further, sEVs isolated from pleural effusion of non-small cell lung cancer (NSCLC) adenocarcinoma patients were analyzed, demonstrating the possibility to profile sEVs from a clinical sample requiring a much lower sample volume.

## 2 Materials and Methods

### 2.1 Reagents

Ultrapure deionized water (resistivity: 18 MΩ·cm) was used throughout the study. Phosphate-buffered saline tablets, avidin from egg-white, streptavidin from *Streptomyces avidinii*, (3-aminopropyl)triethoxysilane (APTES), and glutaraldehyde (GA) were purchased from Sigma-Aldrich Sweden AB. Poly(l-lysine)-*graft*-biotinylated PEG (PLL-*g*-PEG-biotin) was purchased from Nanosoft Polymers. Each subunit was composed of 100 repeating units of PLL with 30% substitution of biotinylated PEG 2000. The copolymer is hereafter referred to as PPB. Silica microcapillaries with an inner diameter of 25 μm were obtained from RISE Acreo AB. Anti-CD9 antibody (MEM-61) (catalog no. NB500-327B) was purchased from Novus Biologicals. Anti-PD-L1 (catalog no. BAF156) and mouse IgG1 isotype control (catalog no. IC002B) antibodies were purchased from Bio-Techne, UK. All of the antibodies were biotin-conjugated.

### 2.2 Electrokinetic Sensing of sEVs

**Figure 1:**
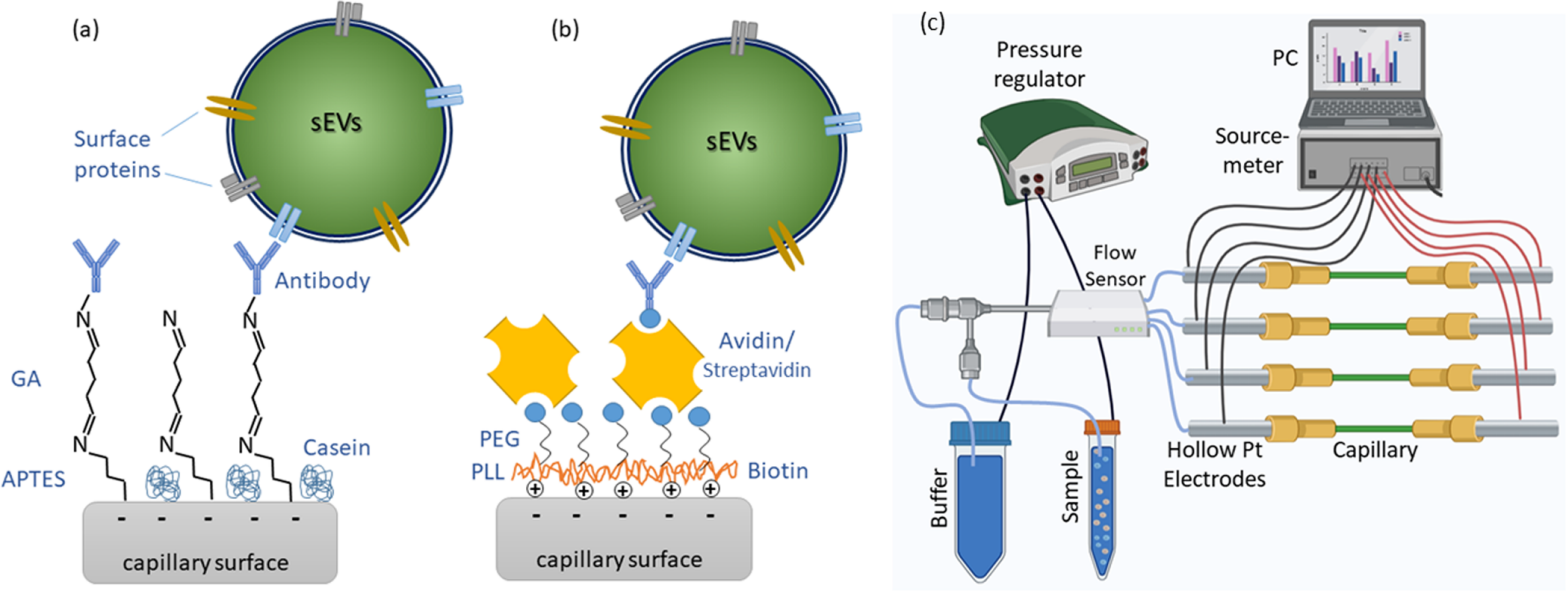
Schematic diagrams of the various functionalization methods used: (a) APTES-GA: the capillary surface was coated with a self-assembled monolayer of APTES, which was linked to the capture antibody via a glutaraldehyde linker. (b) PPB-avidin/streptavidin: the capillary surface was coated with a layer of a PLL-PEG copolymer conjugated with biotin, which was then linked to the capture antibody via an avidin or streptavidin linker. The sEVs were then in both cases detected via the capture antibody. The blocking agents used were (a) casein solution and (b) pluronic F108. Panel (c) shows the experimental setup used in the electrokinetic measurements.

The detection principle applied is based on the streaming current measurement in a commercial microcapillary. The details of the measurement method are described elsewhere.^22,24^ Briefly, the streaming current, generated as a result of a pressure-driven flow of 0.1× PBS through the capillary, was measured using a pair of Pt electrodes. A continuous train of trapezoidal pressure pulses between 1.5 and 3 bar, having a pulse duration of 30 s was used in the method. The pressure pulses were controlled with the help of an Elveflow pressure regulator (OB1). The resulting streaming current pulses (Δ*I*_s_) were measured by a Keithley picoammeter (model no. 2636A), while pressure pulses (Δ*P*) were recorded directly by the pressure regulator. Furthermore, a flow sensor (Elveflow, MSF3) was used to monitor and maintain a stable flow rate through the capillaries. The apparent zeta potential^13^ (ζ*) of the capillary surface was then calculated using the relation
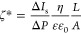
where η and εε_0_ refer to the dynamic viscosity and permittivity of the measurement buffer, respectively, and *L* and *A* refer to the length and cross-sectional area of the capillary, respectively. The measurements involved recording the initial baseline (ζ_i_^*^) and the final baseline after the injection of sEVs (ζ_i_^*^ + Δζ*). The injection of sEVs was done in 1× PBS to maintain the physiological conditions for sEVs, whereas both the baselines were measured in 0.1× PBS to reduce the electrostatic screening. The signal (Δζ*) was hence the change in the baselines as a result of the binding of sEVs to the surface. A multiplexed platform was used for this purpose. A schematic of the measurement setup is shown in Figure 1c.

### 2.3 Capillary Functionalization Protocols

Prior to immobilizing antibodies, the surface of the capillary was chemically modified. The capillaries were first subjected to cleaning by flowing a mixture (5:1:1) of Milli-Q water, 30% H_2_O_2_, and 25% NH_4_OH at 88 °C for 15 min. In this study, three different functionalization strategies were compared, primarily to modify the surface charge (measured in terms of zeta potential) of the functionalized surface. The first method used covalent coupling based on amine-reactive crosslinking chemistry.^25,26^ In this method, a cleaned capillary surface was first coated with APTES by flowing 5% w/v of APTES in 95% ethanol through the capillary for 10 min. Glutaraldehyde (GA), used as a linker between APTES and the capture antibody, was then covalently bound by flowing 1% GA in 1× PBS for 1 h. A detailed description of the method can be found in our previous reports.^22,27^ In the second and third methods, the PPB layer was first electrostatically coupled to the inner surface of a capillary by flowing a solution of PPB in deionized water (0.1 mg/mL) for 30 min. This was followed by conjugation of either avidin or streptavidin, which served as a linker molecule between PPB and biotinylated antibodies. For control measurements, mouse IgG1 isotype control antibody was used instead of the specific capture antibodies. The concentration of the capture/control antibody was 50 μg/mL in 1× PBS and was immobilized for 1 h. In the case of APTES-based functionalization, unreacted aldehyde groups were deactivated by Tris-ethanolamine (0.1 M Tris buffer and 50 mM ethanolamine, pH 9.0) blocking solution for 30 min. This was followed by treatment with 0.05% w/v casein solution for 2 h, to further block nonspecific binding (NSB). Casein treatment was also done on the other fluid junctions in the measuring setup. The NSB suppression in the case of the PPB surface was performed by treatment with pluronic (synperonic) F108 solution, for 15 min on both the capillary as well as other fluid junctions.

## 3 Results

To evaluate the influence of the charge contrast on the detection sensitivity of sEVs, the aspect was first theoretically analyzed. The theoretical predictions were then experimentally validated by comparing three different functionalization strategies (see Section 2), each designed to modulate the surface zeta potential to a large degree. The detection sensitivity of the assay was then tested by profiling surface proteins of sEVs isolated from the cell culture media of a NSCLC cell line. The most sensitive approach, among the different functionalization schemes, was thereafter chosen to further validate the detection principle on sEVs obtained from the PE-fluid samples of NSCLC adenocarcinoma patients.

### 3.1 Simulation to Investigate the Influence of Charge Contrast and Surface Topography

**Figure 2:**
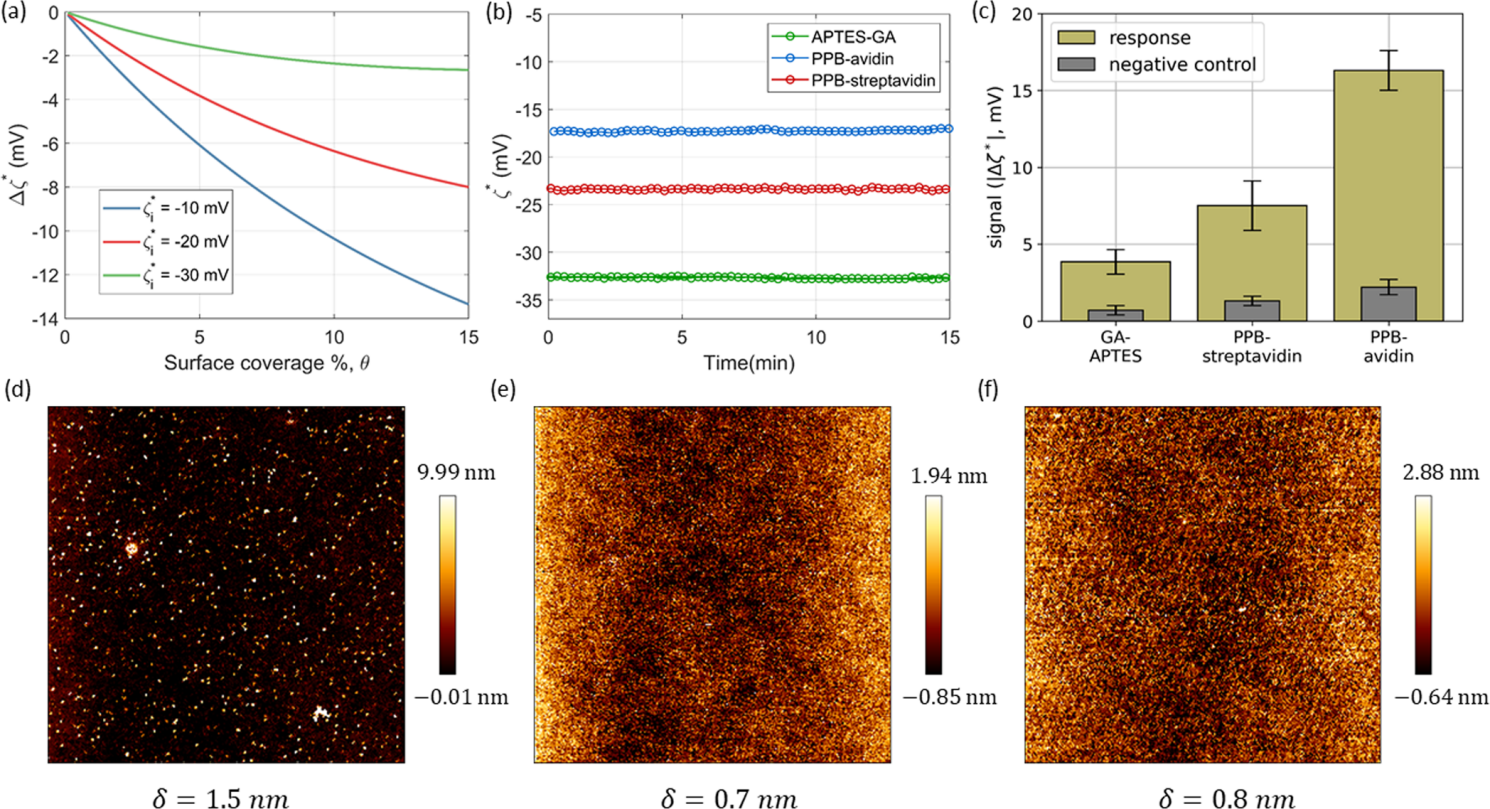
(a) Simulations demonstrating the possibility to enhance the signal by modulating the surface charge, ζ_i_^*^. The signal was simulated for sEVs with ζ_p_ = −30 mV and ζ_i_^*^ = −10, −20, and −30 mV. (b) Initial baselines, ζ_i_^*^, measured for three functionalization methods used: APTES-GA, PPB-avidin, and PPB-streptavidin. (c) Comparison of the signal (Δζ*) obtained on the differently functionalized surface when targeting the extracellular domain of the CD9 membrane protein on sEVs isolated from cell culture media of H1975 cells. The negative control involved mouse IgG1 isotype control antibodies instead of anti-CD9 antibodies. APTES-GA, PPB, and PPB-avidin functionalized surfaces were also studied with AFM (in PBS buffer) to compare their surface roughness. The AFM images for these three surfaces are shown in panels (d)–(f), respectively. The rms roughness (δ) is indicated below each AFM image. The APTES-GA surface shows considerably higher roughness in comparison to the PPB and PPB-avidin surface.

To investigate the influence of charge contrast on the sensitivity, we simulated Δζ* as a function of the surface coverage, θ, for various values of ζ_i_^*^. The simulations were performed using the Adamczyk model,^13,16,28^ which expresses Δζ* as a function of the surface coverage of the bound target (θ), zeta potential of the target particles (ζ_p_), the zeta potential of the capillary surface without any bound sEVs (ζ_i_^*^) given by
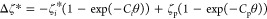
where the parameters *C*_*i*_ and *C*_p_ describe the changes to the macroscopic flow and electrical charge density brought by the bound targets.^28^ The average zeta potential of the target particles (ζ_p_), i.e., sEVs in our case, was assumed to be −30 mV following the reported experimental results^23^ and was kept fixed for the simulations (see Section S6 for further details). Figure 2a shows the plots of Δζ* as a function of θ for various values of ζ_i_^*^. It can be clearly seen that as ζ_i_^*^ changed from −30 to −10 mV, there was a progressive increase in the signal, Δζ*. Decreasing the absolute value of ζ_i_^*^ led to an increase in the charge contrast between the sEVs and the surface, resulting in a stronger response for the same extent of surface coverage of the analyte, i.e., the surface-bound sEVs. The simulated results show that for a surface coverage of 5%, upon changing ζ_i_^*^ from −30 to −20 mV, the signal was enhanced by about 3 times, while a change from −20 to −10 mV was enhanced the signal by about 1.5 times. The model assumes that the surface is ideally smooth before the binding of the targets. In reality, however, the surface has some roughness before the capture of sEVs as a result of the functionalization, which can affect the accuracy of the simulations.^29^

### 3.2 Comparison of Various Surface Functionalization Strategies

After validating the theoretical basis behind the motivation to exploit charge contrast for improved sensitivity, we next characterized the sensor surface prepared with the three different functionalization strategies as described in Section 2.3. First, the surface roughness of differently functionalized surfaces was studied by atomic force microscopy (AFM). For this study, silica-based coverslips were chosen as a substrate since they resemble the surface of a capillary. The results are presented in Figure 2d–f for APTES-GA, PPB, and PPB-avidin-coated surfaces, respectively. To follow the evolution of surface roughness at various steps of the PPB-avidin functionalization strategy, the PPB and PPB-avidin surfaces were analyzed separately. The scale for each image has been adjusted for the best visibility. The rms roughness (δ) was estimated for each of these surfaces. The APTES-GA (δ = 1.5 nm) surface was found to have much higher surface roughness as compared to the PPB (δ = 0.7 nm) and PPB-avidin (δ = 0.8 nm) surface. For characterizing the electrostatic properties of a capillary surface, we then performed the streaming current measurements on a set of microcapillaries functionalized with the three different approaches. The zeta potential of the functionalized capillary surfaces (ζ_i_^*^) estimated from those measurements is presented in Figure 2b. In the case of APTES-GA, after the immobilization of anti-CD9 capture probes, ζ_i_^*^ was found to be −32.8 mV, while for PPB-avidin, it was −16.0 mV, and for PPB-streptavidin, ζ_i_^*^ was −23.3 mV. The absolute values of the signals (|Δζ*|) from the sEV capture in each of the three cases are represented as bar plots in Figure 2c. The |Δζ*| for APTES-GA, PPB-streptavidin, and PPB-avidin were 3.9, 7.4, and 16.3 mV, respectively. Results show that PPB-avidin functionalization led to the strongest signal. Thus, the signal progressively increased as ζ_i_^*^ becomes less negative, which agrees with the simulations (Figure 2a).

### 3.3 Calibration Curve Shows an Improved Limit of Detection

**Figure 3:**
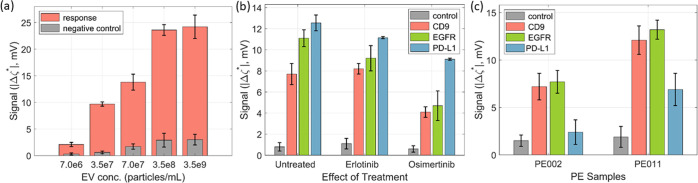
(a) Concentration-dependent response of the sensor when probed with sEVs from the cell culture media of untreated EGFR-mutant NSCLC H1975 cells using biotinylated anti-CD9 antibodies via PPB-based functionalization. The NTA curves of sEVs are shown in Figure S2. The expressions of CD9 and EGFR in these sEVs are previously reported.^30^ (b) Surface expression levels of CD9, EGFR, and PD-L1 for sEVs from cell culture media of H1975 cells prior and post treatments with 1 μM erlotinib or 0.1 μM osimertinib for 48 h. The cell morphology and cell viability of H1975 upon tyrosine kinase inhibitor (TKI) treatment are shown in Figure S3a,b and CD9 expression from Western blot analyses is presented in Figure S3c. (c) sEV samples isolated from PE-fluid of two NSCLC adenocarcinoma patients PE002 and PE011 using the same capturing and affinity reagents as in (b). The control measurements were performed using IgG1 isotype control antibodies.

To further evaluate the improvement in the sensitivity, we estimated the limit of detection (LOD) by measuring the signal as a function of sEV concentration. PPB-avidin was used as the functionalization method as it led to the strongest signal. From the calibration curve thus obtained, the LOD was determined as the concentration of the target corresponding to the minimum detectable signal (MDS) of the sensor. The MDS was taken to be 3× SD of the baseline and was evaluated to be 0.1 mV. Figure 3a shows the signals obtained for different concentrations of sEVs from the cell culture media of H1975 cells captured with anti-CD9 antibodies. The injection of sEVs was done for 2 h in each case. As seen, the signal proportionally increased with the concentration of sEVs. The extent of NSB in each case was estimated by replacing the capture antibodies by isotype control antibodies, and the results showed that NSB remained small in comparison to the signal for the entire range of concentration studied (Figure 3a). From the calibration plot and the MDS level, the LOD was determined to be 4.9 × 10^6^ particles/mL (see Figure S4). This value of LOD with PPB-avidin is approximately 2 orders of magnitude lower than the LOD, which we previously reported using APTES-GA functionalization.^22^

### 3.4 Application: Treatment Monitoring and Liquid Biopsy with Reduced Sample Consumption

For evaluating the sensing platform, we studied sEVs isolated from cell culture media of EGFR-mutant NSCLC cells prior and post EGFR-TKI treatment, i.e., erlotinib or osimertinib. These two EGFR-TKIs are clinically used for NSCLC patients whose tumors are driven by mutations in *EGFR*.^31^ In particular, osimertinib is applied when EGFR harbors the compensatory EGFR mutation T790M in exon 20. There is evidence that PD-L1 is expressed in EGFR-driven NSCLC,^31−33^ and recently, it was also reported that PD-L1 is expressed in sEVs from H1975 cells prior to EGFR-TKI treatment, and it was suggested that PD-L1 plays a role to circumvent immune system attack.^34^ Earlier results have also shown that EGFR-TKI influences PD-L1 expression in EGFR-mutant NSCLC cells.^35^ Therefore, in this study, we focused on EGFR and PD-L1 on sEVs from H1975 cells prior and post erlotinib and osimertinib treatment using our improved sensing method. Figure 3b shows the expression levels of CD9, EGFR, and PD-L1 on sEVs isolated from cell culture media of H1975 cells before and after treatment with erlotinib or osimertinib using our improved sensing method. The doses chosen were 1 μM for erlotinib and 0.1 μM for osimertinib (Figure S3). In the case of erlotinib and in line with literature,^36^ this dose did not cause any effect on cell viability, whereas 0.1 μM osimertinib caused profound effect on cell morphology at 48 h post treatment and reduced viability with about 50% at 72 h post drug addition, when assessed in preparatory experiments (Figure S3B). Figure 3b shows the expression levels of CD9, EGFR, and PD-L1 on the sEVs prior and post treatment. The profiling of CD9 and EGFR was done for a sEV concentration of 3.5 × 10^7^ particles/mL. For this concentration, however, the signal measured for PD-L1 was very small (data not shown). Hence, a 10-fold higher concentration was chosen for profiling PD-L1 to obtain a sufficiently large signal. When comparing sEVs from untreated and erlotinib treated cells, the CD9 level remained nearly unchanged, whereas EGFR and PD-L1 exhibited reduced expression of about 20 and 10%, respectively. The western blot analyses of CD9 expression in sEVs and H1975 cells are shown in Figure S3c. The reduction in EGFR may be a result of a modest effect of erlotinib on H1975 cells. This could have, to some extent, targeted the EGFR-expressing cells, reducing the sEVs that express EGFR. In line with earlier reports on EGFR-mutant NSCLC cells,^35^ erlotinib reduced PD-L1 expression on sEVs as monitored by our sensor, but as H1975 cells were unresponsive to erlotinib the reduction was rather modest. In the case of the osimertinib treatment, cells resulted in a stronger decline in the expression level of all three proteins. In particular, CD9 and EGFR showed a sharp decrease: by about 50 and 60%, respectively, whereas PD-L1 expression was reduced by about 30% (Figure 3b).

The prospect of a higher sensitivity of a sensor is especially important in a clinical setting as the quantity of samples available from patients may be limited. This necessitates the profiling of sEV surface markers using a low sample volume. Therefore, the improved technique was also tested on sEVs isolated from PE-fluid samples of two NSCLC patients, PE002 and PE011, having an ALK and EGFR-driven tumor, respectively (Table 1). The results presented in Figure 3c indicate that CD9, EGFR, and PD-L1 have higher expression levels in sEVs from PE011 as compared to PE002. These trends match with our earlier results using the APTES-GA functionalization^24^ as well as with immuno-PCR and western blot analyses.^30^ However, the present investigation has been carried out with sEV concentrations that were 100 times lower in the case of CD9 and EGFR, and 10 times lower in the case of PD-L1 in comparison to that in our previous studies.

**Table 1:** Details of H1975 and PE-Fluid Isolated sEVs for Validating the Improved Method for Profiling the Membrane Proteins of sEVs^a^

sample	treatment	tumor stage	genomic alteration	feature
H1975 cell line	untreated		EGFR exon 21, L858R, exon 20 T790M	
	erlotinib			resistant^36^ (see Figure S3)
	osimertinib			responsive (see Figure S3)
PE002	ALK TKI crizotinib	T2aN0M1a	EML4-ALK variant 3 (a/b)	responsive
PE011	EGFR-TKI erlotinib	T4N2M1a	EGFR exon 21, L858R	progressive

a The cell line-derived sEVs were used to monitor the effects of TKI treatment, whereas plural effusion (PE) samples were used to determine the possibility to analyze sEVs from the complex patients’ sample.

## 4 Discussion

One of the main novelties of this study lies on the modulation of surface charge through a generic chemical functionalization strategy to achieve a higher sensitivity. Indeed, sensitivity is one of the most important criteria irrespective of sensing modalities and therefore has received significant research interest. In the case of surface-based sensors utilizing electrical/electrostatic effects for signal transduction, several attempts to enhance their sensitivity have been made previously. For example, in the case of electrochemical sensing, different methods such as enzyme-assisted amplification strategies,^37^ nanoparticle-mediated approach,^38^ and surface charge modulation approach using peptide nucleic acid for DNA detection^39^ have been reported. The electrostatic influence on the sensitivity has also been studied for ion-sensitive field-effect transistor (ISFETs)-based sensors.^40^ For ISFETs, the sensitivity was found to depend on the isoelectric point of the target analyte.^41^ The surface charge modulation strategies have also been explored for nanopore-based sensing.^42^ Clearly, the fundamental principle of electric charge-based signal enhancement has been explored before. However, a strategy for deliberate modulation of surface charge for signal enhancement has rarely been systematically addressed and, to the best of our knowledge, has never been investigated for streaming current-based biosensing, although the influence of surface charge on streaming current has been known and investigated in a number of studies by us and others.^13,15,16^ It is expected that the chemical composition of the surface layer used for the immobilization of biological receptor will ultimately determine the electrostatic property of the surface. In the case of biosensing using a streaming current-based method, the conjugation of target molecules to a surface changes the electrostatic property of the surface, which is detected by monitoring the changes in the streaming current. Accordingly, the charge contrast between the surface and the target is expected to strongly influence the detection sensitivity. Therefore, for a given target, the optimization of the electrostatic property of the surface is one of the most important design criteria for improving the detection sensitivity. Biological analytes, such as proteins, amino acids, etc., have a well-defined isoelectric point, and thus, the principle of charge contrast can be exploited for an optimum sensor design achieving high sensitivity. The principle is equally applicable for sEVs despite the fact that they are heterogeneous in terms of their molecular composition^43^ and hence their electrostatic properties. This is because sEVs retain a large net negative charge at physiological pH due to the presence of deprotonated COO^-^ groups of proteins, acidic sugars, and/or exposed phosphate groups of DNA.^23^ Therefore, a functionalization strategy that makes the surface zeta potential less negative or more positive is preferable. The argument is clearly validated from the theoretical perspective in this study which predicts a stronger signal with a less negative ζ_i_^*^.

The silica surface has an isoelectric point of ∼3.9.^44^ Therefore, the surface remains highly negative at the buffer pH used in this study. The zeta potential of a clean capillary was measured to be −68.6 mV. Using the Gouy–Chapman equation, the effective surface charge density (σ_eff_) could be extracted (see Section S2 for details) for the surface at various stages of the functionalization and is presented in Table 2. In the case of the clean capillary, it was estimated to be −5.1 × 10^–3^ e/nm.^2^ The coating of APTES-GA on the capillary surface partly screens the negative charges producing a net σ_eff_ = −2.0 × 10^–3^ e/nm^2^ for the CD9 antibody conjugated surface in our work. In contrast, PLL in the PBS buffer (pH = 7.4) carries a strong positive charge on the ε-amine of its side chains.^45^ As a result, PPB can be easily and conformally coated on a silica surface by exploiting their strong electrostatic interaction.^46,47^By partially screening the negative charges of silica, as well as exposing its own positive charges, the PPB coating results in a net σ_eff_ of −1.6 × 10^–3^ e/nm^2^. Moreover, coating avidin (pI ∼10 ^47^) as a linker between a PPB layer and antibody introduces more positive charges on the surface and, therefore, further reduces σ_eff_ to −8.1 × 10^–4^ e/nm^2^. In our study, we also used streptavidin as a linker instead of avidin. This allowed us to modulate σ_eff_ to an intermediate level of 1.3 × 10^–3^ e/nm^2^ since the pI of streptavidin is ∼5.^48^ It is important to highlight an assumption used in estimating the values of σ_eff_. The Gouy–Chapman equation assumes that the surface is arbitrarily flat.^49^ A clean capillary surface can be assumed to be flat in the length scale of the Debye screening length (2.3 nm in this case^13^). Upon chemical functionalization, its roughness is expected to increase. However, AFM measurements show that this roughness is still very small. As presented in Figure 2d,f, the rms roughness of PPB-avidin- and APTES-GA-coated surfaces were measured to be 0.8 and 1.5 nm, respectively. Hence, the assumption is justified. This method cannot be used, however, to estimate the σ_eff_ of the surface after the capture of sEVs, whose mean diameter was about 200 nm (see NTA data in Figure S2), and would hence lead to a considerable increase in the surface roughness. Moreover, the significantly lower roughness of the PPB-avidin-coated surface in comparison to the APTES-GA-coated surface is also partly responsible for the higher sensitivity of the sensor.^13,29^ It must be pointed out that it was verified separately with optical measurements that the difference in sensitivity from the APTES-GA-based and PPB-avidin-based functionalization strategies does not arise due to the different extent of surface coverage of the sEVs (see Section S1, Supporting Information for details). The demonstrated strategy for surface charge modulation is generic in nature and can also be utilized for other sensor types, e.g., ISFETs, nanopores, etc.

**Table 2:** ζ_i_^*^ and σ_eff_ of Surfaces at Various Stages of Functionalization^a^

surface	ζ_i_^*^ (mV)	σ_eff_ (10^–3^ e/nm^2^)
bare silica	–68.6	–5.2
GA-APTES	–32.5	–2.0
GA-APTES-anti-CD9	–32.8	–2.0
PPB	–26.3	–1.6
PPB-avidin	–14.1	–0.8
PPB-avidin-anti-CD9	–16.0	–0.9
PPB-streptavidin	–20.7	–1.2
PPB-streptavidin-anti-CD9	–23.3	–1.4

a Clearly, PPB-avidin leads to the least negatively charged surface among the three functionalization schemes tested.

The expected outcome of the increased charge contrast is visible in the sensor response to sEV detection by the CD9 membrane protein. In good agreement with the simulation, the signal obtained with PPB-avidin was enhanced by ∼5 fold in comparison to GA-APTES, whereas that obtained with PPB-streptavidin had a 2-fold enhancement (Figure 2c). Due to the same reason, the responses of the negative control in each case also followed the same trend as the signal. To the best of our knowledge, the LOD obtained with this improved method is the highest reported thus far for the streaming current-based sensing method. Apart from the clear advantage of signal enhancement, PPB-based functionalization also offers several other benefits. APTES and GA are both toxic, which is a serious limitation. Besides, the functionalization strategy involves a number of different steps (see Section 3), thereby requiring a relatively long preparation time (approximately 6 h). In comparison, PPB-based strategy requires a shorter functionalization time (about 3 h). In this case, the PLL backbone interacts electrostatically with the silica substrate. The side chains can be modified with different functional groups adding other benefits. In the present study, PEG-grafted PLL was used since it has been demonstrated to be extremely effective against reducing NSB.^50^

The improved sensitivity of the detection method is beneficial for their clinical application as less amount of sample is required. This is demonstrated in the present study using sEVs from the PE-fluid of two NSCLC patients with EGFR mutation (PE011) or ALK-fusion (PE002). Compared to our previous study,^24^ the PPB-avidin functionalization allowed us to obtain the results on CD9, EGFR, and PD-L1 expression levels on these samples with a significantly lower (∼1/100th and 1/10th, respectively) sample volume. Moreover, the obtained results with respect to EGFR and CD9 expression are also in line with the results from immuno-PCR analyses of the same samples.^30^ It is however important to point out that the signals obtained against different surface proteins for a particular sample are not representative of the relative expression levels of the proteins for that sample. This is not only because of the different ζ_i_^*^ resulting from the different capture probe against each protein profiled but also due to the fact that the affinity of the capture probe to its corresponding protein also varies. Hence, for comparison, the signals from only the same surface protein should be compared across different samples and not with the signals obtained for other surface proteins of the same sample. The study also opens up the possibility for this method to be used at point-of-care for treatment monitoring of cancer patients as the streaming current method is portable and lab-on-chip compatible.

Recently, there has been a lot of research interest toward using sEVs for cancer treatment monitoring.^21,51−54^ This stems from the possibility of extracting sEVs from a wide range of body fluids such as plasma, serum, pleural effusion fluid, cerebrospinal fluid, urine, saliva, etc., for performing noninvasive liquid biopsy in a real time and dynamic manner.^51^ As genomic and molecular characterization of tumors including NSCLC have allowed for precision cancer medicine treatment regimen, e.g., targeting aberrant growth factor receptors by TKIs or restoring the attack of the immune system against the tumor by PD-1/PD-L1 antibodies, the need for noninvasive monitoring as those presented by analyses of sEVs in liquid biopsies has increased.^21^ Thus, in response to EGFR-TKI^54^ as well as to the immune therapy^34^ analyses of the sEV surface or cargo, biomolecules have been demonstrated to be feasible to follow the treatment response of individual patients. Our measurements show that H1975 cells are rather unresponsive to erlotinib (Figure 3b). This is due to the presence of T790M mutation, which hampers its binding to the EGFR-kinase domain.^55^ One could, however, expect to have some effect and hence after 48 h of treatment, a reduction in the EGFR expression level could be seen in sEVs (Figure S3). Although the expression of PD-L1 on sEVs from untreated H1975 cells agrees well with a previous report,^34^ the reduced expression of PD-L1 after erlotinib treatment needs further mechanistic evaluation. On the other hand, osimertinib treatment caused about a 50% reduction in H1975 cell viability (Figure S3). Our measurements reveal that this is accompanied by a larger reduction in the expression levels of CD9, EGFR, and PD-L1 membrane proteins of sEVs (Figure 3b).

For clinical utility, such sEV analyses require not only the capacity to use a small sample volume but also sensors that are sensitive enough to monitor also sEV surface proteins that have a low expression level. This is illustrated in our study of PD-L1, which generated a signal just above the MDS level for the sample PE002 in our previous report^24^ when the concentration of the sEVs used for the measurement was 3.5 × 10^9^ particles/mL. In contrast, with the PPB-avidin functionalization approach, a much stronger signal could be obtained despite using 1/10th of the concentration used previously. Our results also show that the sensor can monitor alterations in EGFR and PD-L1 expression levels after in vitro treatment of EGFR-mutant NSCLC cells with EGFR-TKI erlotinib or osimertinib. Here, the path ahead is to monitor sEVs from plasma or serum of NSCLC patients during a treatment course to further substantiate the clinical usefulness of our improved sensor. Another benefit from using a PPB-based functionalization is the ease of regenerating the sensor surface to have reusable sensors. PLL adsorption on the silica surface has been shown to be reversible, the desorption of PLL being carried out via a highly basic buffer (pH ∼12).^19^ This opens the possibility to make the sensor reusable.

## 5 Conclusions

In summary, the electrostatic charge contrast was shown to be a crucial criterion in selecting the functionalization strategy of electrokinetic biosensors. This was demonstrated using three different functionalization approaches for modulating the zeta potential of the sensor surface. The highest sensitivity was shown by the sensor with the largest charge contrast between the sensor surface and the target. The limit of detection (LOD) varied across 2 orders of magnitude, reaching a value of 4.9 × 10^6^ particles/mL for the optimal functionalization approach. This approach was then used for profiling the membrane protein of sEVs isolated from NSCLC H1975 cell culture media after treatment with the EGFR-TKIs erlotinib and osimertinib, as well as sEVs obtained from PE-fluid of NSCLC adenocarcinoma patients. The results verify the trends obtained by other studies, but, importantly, they were obtained with a much lower sample volume as compared to previous measurements. This shows the potential of this sensing platform as a promising tool for treatment monitoring in NSCLC and other tumor malignancies.

## Supporting Information Available

Details of the fluorescence measurements, effective charge density calculations, details of the NTA measurements, EGFR-TKI Erlotinib and Osimertinib responses in H1975 cells, estimation of the LOD, details of the simulation parameters, sEV isolation and characterization, cell morphology and cell viability analyses post EGFR-TKI treatment and preparation of the biotinylated EGFR-binding antibody (PDF)

## References

[ref1] Chen Y.; Ren R.; Pu H.; Guo X.; Chang J.; Zhou G.; Mao S.; Kron M.; Chen J. Field-Effect Transistor Biosensor for Rapid Detection of Ebola Antigen. Sci. Rep. 2017, 7, 10974 10.1038/s41598-017-11387-7. 28887479 PMC5591202

[ref2] Bhattacharjee M.; Nemade H. B.; Bandyopadhyay D. Nano-Enabled Paper Humidity Sensor for Mobile Based Point-of-Care Lung Function Monitoring. Biosens. Bioelectron. 2017, 94, 544–551. 10.1016/J.BIOS.2017.03.049. 28351016

[ref3] Chen X.; Guo Z.; Yang G.-M.; Li J.; Li M.-Q.; Liu J.-H.; Huang X.-J. Electrical Nanogap Devices for Biosensing. Mater. Today 2010, 13, 28–41. 10.1016/S1369-7021(10)70201-7.

[ref4] Katira P.; Hess H. Two-Stage Capture Employing Active Transport Enables Sensitive and Fast Biosensors. Nano Lett. 2010, 10, 567–572. 10.1021/NL903468P. 20055432 PMC2819759

[ref5] Sitt A.; Hess H. Directed Transport by Surface Chemical Potential Gradients for Enhancing Analyte Collection in Nanoscale Sensors. Nano Lett. 2015, 15, 3341–3350. 10.1021/ACS.NANOLETT.5B00595. 25817944

[ref6] Rahman M. M.; Rana M. M.; Rahman M. S.; Anower M. S.; Mollah M. A.; Paul A. K. Sensitivity Enhancement of SPR Biosensors Employing Heterostructure of PtSe2 and 2D Materials. Opt. Mater. 2020, 107, 110123 10.1016/J.OPTMAT.2020.110123.

[ref7] van den Berg A.; Bergveld P.; Reinhoudt D. N.; Sudhölter E. J. Sensitivity Control of ISFETs by Chemical Surface Modification. Sens. Actuators 1985, 8, 129–148. 10.1016/0250-6874(85)87010-4.

[ref8] Yang Y. L.; Lo L. H.; Huang I. Y.; Chen H. J. H.; Huang W. S.; Huang S. R. S. Improvement of Polyimide Capacitive Humidity Sensor by Reactive Ion Etching and Novel Electrode Design. Proc. IEEE Sens. 2002, 1, 511–514. 10.1109/ICSENS.2002.1037147.

[ref9] Rivadeneyra A.; Fernández-Salmerón J.; Banqueri J.; López-Villanueva J. A.; Capitan-Vallvey L. F.; Palma A. J. A Novel Electrode Structure Compared with Interdigitated Electrodes as Capacitive Sensor. Sens. Actuators, B 2014, 204, 552–560. 10.1016/J.SNB.2014.08.010.

[ref10] Gunda N. S. K.; Singh M.; Norman L.; Kaur K.; Mitra S. K. Optimization and Characterization of Biomolecule Immobilization on Silicon Substrates Using (3-Aminopropyl)Triethoxysilane (APTES) and Glutaraldehyde Linker. Appl. Surf. Sci. 2014, 305, 522–530. 10.1016/j.apsusc.2014.03.130.

[ref11] Liu L.; Etika K. C.; Liao K. S.; Hess L. A.; Bergbreiter D. E.; Grunlan J. C. Comparison of Covalently and Noncovalently Functionalized Carbon Nanotubes in Epoxy. Macromol. Rapid Commun. 2009, 30, 627–632. 10.1002/marc.200800778. 21706651

[ref12] Zhang Y.; Chu C.-W.; Ma W.; Takahara A. Functionalization of Metal Surface via Thiol–Ene Click Chemistry: Synthesis, Adsorption Behavior, and Postfunctionalization of a Catechol- and Allyl-Containing Copolymer. ACS Omega 2020, 5, 7488–7496. 10.1021/ACSOMEGA.0C00259. 32280892 PMC7144137

[ref13] Sahu S. S.; Stiller C.; Cavallaro S.; Karlström A. E.; Linnros J.; Dev A. Influence of Molecular Size and Zeta Potential in Electrokinetic Biosensing. Biosens. Bioelectron. 2020, 152, 112005 10.1016/j.bios.2020.112005. 32056733

[ref14] Sahu S. S.; Stiller C.; Gomero E. P.; Nagy Á.; Karlström A. E.; Linnros J.; Dev A. Electrokinetic Sandwich Assay and DNA Mediated Charge Amplification for Enhanced Sensitivity and Specificity. Biosens. Bioelectron. 2021, 176, 112917 10.1016/j.bios.2020.112917. 33421763

[ref15] Michelmore A. P.; Hayes R. A. The Effect of Deposition of Negatively Charged Particles on the Electrokinetic Behaviour of Oppositely Charged Surfaces. PhysChemComm 2000, 3, 24 10.1039/b001277g.

[ref16] Sadlej K.; Wajnryb E.; Bawzdziewicz J.; Ekiel-Jeewska M. L.; Adamczyk Z. Streaming Current and Streaming Potential for Particle Covered Surfaces: Virial Expansion and Simulations. J. Chem. Phys. 2009, 130, 144706 10.1063/1.3103545. 19368464

[ref17] Wasilewska M.; Adamczyk Z. Fibrinogen Adsorption on Mica Studied by AFM and in Situ Streaming Potential Measurements. Langmuir 2011, 27, 686–696. 10.1021/la102931a. 21155546

[ref18] Morga M.; Adamczyk Z.; Gödrich S.; Oćwieja M.; Papastavrou G. Monolayers of Poly-l-Lysine on Mica - Electrokinetic Characteristics. J. Colloid Interface Sci. 2015, 456, 116–124. 10.1016/j.jcis.2015.05.044. 26115031

[ref19] Lepoitevin M.; Jamilloux B.; Bechelany M.; Balanzat E.; Janot J. M.; Balme S. Fast and Reversible Functionalization of a Single Nanopore Based on Layer-by-Layer Polyelectrolyte Self-Assembly for Tuning Current Rectification and Designing Sensors. RSC Adv. 2016, 6, 32228–32233. 10.1039/c6ra03698h.

[ref20] Serrano Â.; Zürcher S.; Tosatti S.; Spencer N. D. Imparting Nonfouling Properties to Chemically Distinct Surfaces with a Single Adsorbing Polymer: A Multimodal Binding Approach. Macromol. Rapid Commun. 2016, 37, 622–629. 10.1002/marc.201500683. 26858017

[ref21] Santarpia M.; Liguori A.; D’Aveni A.; Karachaliou N.; Gonzalez-Cao M.; Daffinà M. G.; Lazzari C.; Altavilla G.; Rosell R. Liquid Biopsy for Lung Cancer Early Detection. J. Thorac. Dis. 2018, S882–S897. 10.21037/jtd.2018.03.81. 29780635 PMC5945693

[ref22] Cavallaro S.; Horak J.; Hååg P.; Gupta D.; Stiller C.; Sahu S. S.; Görgens A.; Gatty H. K.; Viktorsson K.; El-Andaloussi S.; Lewensohn R.; Eriksson Karlström A.; Linnros J.; Dev A. Label-Free Surface Protein Profiling of Extracellular Vesicles by an Electrokinetic Sensor. ACS Sens. 2019, 4, 1399–1408. 10.1021/acssensors.9b00418. 31020844

[ref23] Midekessa G.; Godakumara K.; Ord J.; Viil J.; Lättekivi F.; Dissanayake K.; Kopanchuk S.; Rinken A.; Andronowska A.; Bhattacharjee S.; Rinken T.; Fazeli A. Zeta Potential of Extracellular Vesicles: Toward Understanding the Attributes That Determine Colloidal Stability. ACS Omega 2020, 5, 16701–16710. 10.1021/acsomega.0c01582. 32685837 PMC7364712

[ref24] Cavallaro S.; Hååg P.; Sahu S. S.; Berisha L.; Kaminskyy V. O.; Ekman S.; Lewensohn R.; Linnros J.; Viktorsson K.; Dev A. Multiplexed Electrokinetic Sensor for Detection and Therapy Monitoring of Extracellular Vesicles from Liquid Biopsies of Non-Small-Cell Lung Cancer Patients. Biosens. Bioelectron. 2021, 193, 113568 10.1016/j.bios.2021.113568. 34428672

[ref25] Rusmini F.; Zhong Z.; Feijen J. Protein Immobilization Strategies for Protein Biochips. Biomacromolecules 2007, 1775–1789. 10.1021/bm061197b. 17444679

[ref26] Jonkheijm P.; Weinrich D.; Schröder H.; Niemeyer C. M.; Waldmann H. Chemical Strategies for Generating Protein Biochips. Angew. Chem., Int. Ed. 2008, 9618–9647. 10.1002/anie.200801711. 19025742

[ref27] Dev A.; Horak J.; Kaiser A.; Yuan X.; Perols A.; Björk P.; Karlström A. E.; Kleimann P.; Linnros J. Electrokinetic Effect for Molecular Recognition: A Label-Free Approach for Real-Time Biosensing. Biosens. Bioelectron. 2016, 82, 55–63. 10.1016/j.bios.2016.03.060. 27040942

[ref28] Adamczyk Z.; Sadlej K.; Wajnryb E.; Nattich M.; Ekiel-Jezewska M. L.; Bławzdziewicz J. Streaming Potential Studies of Colloid, Polyelectrolyte and Protein Deposition. Adv. Colloid Interface Sci. 2010, 153, 1–29. 10.1016/j.cis.2009.09.004. 19926067

[ref29] Ekiel-Jeewska M. L.; Adamczyk Z.; Blawzdziewicz J. Streaming Current and Effective ζ-Potential for Particle-Covered Surfaces with Random Particle Distributions. J. Phys. Chem. C 2019, 123, 3517–3531. 10.1021/acs.jpcc.8b10068. 19368464

[ref30] Stiller C.; Viktorsson K.; Gomero E. P.; Hååg P.; Arapi V.; Kaminskyy V. O.; Kamali C.; De Petris L.; Ekman S.; Lewensohn R.; Karlström A. E. Detection of Tumor-associated Membrane Receptors on Extracellular Vesicles from Non-small Cell Lung Cancer Patients via Immuno-pcr. Cancers 2021, 13, 1–21. 10.3390/cancers13040922. PMC7926549 33671772

[ref31] König D.; Prince S. S.; Rothschild S. I. Targeted Therapy in Advanced and Metastatic Non-Small Cell Lung Cancer. An Update on Treatment of the Most Important Actionable Oncogenic Driver Alterations. Cancers 2021, 1–37. 10.3390/cancers13040804. PMC7918961 33671873

[ref32] Abdel Karim N.; Kelly K. Role of Targeted Therapy and Immune Checkpoint Blockers in Advanced Non-Small Cell Lung Cancer: A Review. Oncologist 2019, 24, 1270–1284. 10.1634/theoncologist.2018-0112. 30914465 PMC6738296

[ref33] Kang M.; Park C.; Kim S. H.; Yoon S. W.; Suh K. J.; Kim Y. J.; Ock C. Y.; Kim M.; Keam B.; Kim T. M.; Kim D. W.; Heo D. S.; Lee J. S. Programmed Death-Ligand 1 Expression Level as a Predictor of EGFR Tyrosine Kinase Inhibitor Efficacy in Lung Adenocarcinoma. Transl. Lung Cancer Res. 2021, 10, 699–711. 10.21037/tlcr-20-893. 33718015 PMC7947423

[ref34] Kim D. H.; Kim H. R.; Choi Y. J.; Kim S. Y.; Lee J. E.; Sung K. J.; Sung Y. H.; Pack C. G.; Jung M. k.; Han B.; Kim K.; Kim W. S.; Nam S. J.; Choi C. M.; Yun M.; Lee J. C.; Rho J. K. Exosomal PD-L1 Promotes Tumor Growth through Immune Escape in Non-Small Cell Lung Cancer. Exp. Mol. Med. 2019, 51, 1–13. 10.1038/s12276-019-0295-2. PMC6802663 31399559

[ref35] Zhang N.; Zeng Y.; Du W.; Zhu J.; Shen D.; Liu Z.; Huang J.-A. The EGFR Pathway Is Involved in the Regulation of PD-L1 Expression via the IL-6/JAK/STAT3 Signaling Pathway in EGFR-Mutated Non-Small Cell Lung Cancer. Int. J. Oncol. 2016, 49, 1360–1368. 10.3892/IJO.2016.3632. 27499357

[ref36] Pao W.; Miller V. A.; Politi K. A.; Riely G. J.; Somwar R.; Zakowski M. F.; Kris M. G.; Varmus H. Acquired Resistance of Lung Adenocarcinomas to Gefitinib or Erlotinib Is Associated with a Second Mutation in the EGFR Kinase Domain. PLoS Med. 2005, 2, e73 10.1371/journal.pmed.0020073. 15737014 PMC549606

[ref37] Sun J.; Gan Y.; Liang T.; Zhou S.; Wang X.; Wan H.; Wang P. Signal Enhancement of Electrochemical DNA Biosensors for the Detection of Trace Heavy Metals. Curr. Opin. Electrochem. 2019, 17, 23–29. 10.1016/J.COELEC.2019.04.007.

[ref38] Upan J.; Youngvises N.; Tuantranont A.; Karuwan C.; Banet P.; Aubert P.-H.; Jakmunee J. A Simple Label-Free Electrochemical Sensor for Sensitive Detection of Alpha-Fetoprotein Based on Specific Aptamer Immobilized Platinum Nanoparticles/Carboxylated-Graphene Oxide. Sci. Rep. 2021, 11, 1–9. 10.1038/s41598-021-93399-y. 34234187 PMC8263621

[ref39] Won B. Y.; Yoon H. C.; Park H. G. Enzyme-Catalyzed Signal Amplification for Electrochemical DNA Detection with a PNA-Modified Electrode. Analyst 2008, 133, 100–104. 10.1039/B712638G. 18087620

[ref40] Ditshego N. M. J.; Ghazali N. A. B.; Ebert M.; Sun K.; Zeimpekis I.; Ashburn P.; De Planque M. R. R.; Chong H. M. H. ZnO Nanowire-FET for Charge-Based Sensing of Protein Biomolecules. In 2015 IEEE 15th International Conference on Nanotechnology (IEEE-NANO), 2015; pp 801–804.

[ref41] McKinnon W. R.; Landheer D.; Aers G. Sensitivity of Field-Effect Biosensors to Charge, PH, and Ion Concentration in a Membrane Model. J. Appl. Phys. 2008, 104, 124701 10.1063/1.3050329.

[ref42] Cai S. L.; Cao S. H.; Zheng Y.; Zhao S.; Yang J. L.; Li Y. Q. Surface Charge Modulated Aptasensor in a Single Glass Conical Nanopore. Biosens. Bioelectron. 2015, 71, 37–43. 10.1016/J.BIOS.2015.04.002. 25884732

[ref43] Cavallaro S.; Pevere F.; Stridfeldt F.; Görgens A.; Paba C.; Sahu S. S.; Mamand D. R.; Gupta D.; El Andaloussi S.; Linnros J.; Dev A. Multiparametric Profiling of Single Nanoscale Extracellular Vesicles by Combined Atomic Force and Fluorescence Microscopy: Correlation and Heterogeneity in Their Molecular and Biophysical Features. Small 2021, 17, 2008155 10.1002/smll.202008155. 33682363

[ref44] Cuddy M. F.; Poda A. R.; Brantley L. N. Determination of Isoelectric Points and the Role of PH for Common Quartz Crystal Microbalance Sensors. ACS Appl. Mater. Interfaces 2013, 5, 3514–3518. 10.1021/am400909g. 23611583

[ref45] Godbey W. T. An Introduction to Biotechnology: The Science, Technology and Medical Applications; Elsevier, 2014.

[ref46] Iqbal A.; Chapin J. C.; Mehdizadeh E.; Rahafrooz A.; Purse B. W.; Pourkamali S. Real-Time Bio-Sensing Using Micro-Channel Encapsulated Thermal- Piezoresistive Rotational Mode Disk Resonators. Proc. IEEE Sens. 2012, 10.1109/ICSENS.2012.6411385.

[ref47] Bruch R. C.; White H. B. Compositional and Structural Heterogeneity of Avidin Glycopeptides. Biochemistry 1982, 21, 5334–5341. 10.1021/bi00264a033. 6816268

[ref48] Chaiet L.; Wolf F. J. The Properties of Streptavidin, a Biotin-Binding Protein Produced by Streptomycetes. Arch. Biochem. Biophys. 1964, 106, 1–5. 10.1016/0003-9861(64)90150-X. 14217155

[ref49] Ge Z.; Wang Y. Estimation of Nanodiamond Surface Charge Density from Zeta Potential and Molecular Dynamics Simulations. J. Phys. Chem. B 2017, 121, 3394–3402. 10.1021/acs.jpcb.6b08589. 28423901

[ref50] Charles P. T.; Stubbs V. R.; Soto C. M.; Martin B. D.; White B. J.; Taitt C. R. Reduction of Non-Specific Protein Adsorption Using Poly(Ethylene) Glycol (PEG) Modified Polyacrylate Hydrogels in Immunoassays for Staphylococcal Enterotoxin B Detection. Sensors 2009, 9, 645–655. 10.3390/s90100645. 22389622 PMC3280768

[ref51] Stevic I.; Buescher G.; Ricklefs F. L. Monitoring Therapy Efficiency in Cancer through Extracellular Vesicles. Cells 2020, 130 10.3390/cells9010130. PMC7017260 31935901

[ref52] Wang J.; Wuethrich A.; Sina A. A. I.; Lane R. E.; Lin L. L.; Wang Y.; Cebon J.; Behren A.; Trau M. Tracking Extracellular Vesicle Phenotypic Changes Enables Treatment Monitoring in Melanoma. Sci. Adv. 2020, 6, eaax3223 10.1126/sciadv.aax3223. 32133394 PMC7043913

[ref53] Liang K.; Liu F.; Fan J.; Sun D.; Liu C.; Lyon C. J.; Bernard D. W.; Li Y.; Yokoi K.; Katz M. H.; Koay E. J.; Zhao Z.; Hu Y. Nanoplasmonic Quantification of Tumour-Derived Extracellular Vesicles in Plasma Microsamples for Diagnosis and Treatment Monitoring. Nat. Biomed. Eng. 2017, 1, 21 10.1038/s41551-016-0021. PMC5543996 28791195

[ref54] Krug A. K.; Enderle D.; Karlovich C.; Priewasser T.; Bentink S.; Spiel A.; Brinkmann K.; Emenegger J.; Grimm D. G.; Castellanos-Rizaldos E.; Goldman J. W.; Sequist L. V.; Soria J. C.; Camidge D. R.; Gadgeel S. M.; Wakelee H. A.; Raponi M.; Noerholm M.; Skog J. Improved EGFR Mutation Detection Using Combined Exosomal RNA and Circulating Tumor DNA in NSCLC Patient Plasma. Ann. Oncol. 2018, 29, 700–706. 10.1093/annonc/mdx765. 29216356 PMC5889041

[ref55] Tang Z.; Du R.; Jiang S.; Wu C.; Barkauskas D. S.; Richey J.; Molter J.; Lam M.; Flask C.; Gerson S.; Dowlati A.; Liu L.; Lee Z.; Halmos B.; Wang Y.; Kern J. A.; Ma P. C. Dual MET-EGFR Combinatorial Inhibition against T790M-EGFR-Mediated Erlotinib-Resistant Lung Cancer. Br. J. Cancer 2008, 99, 911–922. 10.1038/sj.bjc.6604559. 19238632 PMC2538758

