# Supplementary material for: Exploiting Electrostatic Interaction for Highly Sensitive Detection of Tumor-Derived Extracellular Vesicles by an Electrokinetic Sensor
Source: ACS Appl Mater Interfaces. 2021 Sep 2;13(36):42513–21. doi: 10.1021/acsami.1c13192 (PMC8447189; doi:10.1021/acsami.1c13192)
Supplement: Supplementary file 1 — am1c13192_si_001.pdf [file am1c13192_si_001.pdf]

Supporting information for:

Exploiting electrostatic interaction for highly sensitive detection of tumor-derived extracellular vesicles by an electrokinetic Sensor

Siddharth Sourabh Sahu[1,*], Sara Cavallaro[2], Petra Hååg[3], Ábel Nagy[4], Amelie Eriksson Karlström[4], Rolf Lewensohn[3,5], Kristina Viktorsson[3], Jan Linnros[2], Apurba Dev[1,2,*]

[1]Department of Electrical Engineering, The Ångström Laboratory, Uppsala University, 75121 Uppsala, Sweden.

[2]Department of Applied Physics, School of Engineering Sciences, KTH Royal Institute of Technology, 10691 Stockholm, Sweden.

[3] Department of Oncology-Pathology, Karolinska Institutet, 17164 Stockholm, Sweden.

[4]Department of Protein Science, School of Chemistry, Biotechnology, and Health (CBH), KTH Royal Institute of Technology, 10691 Stockholm, Sweden.

[5]Theme Cancer, Patient Area Head and Neck, Lung, and Skin, Karolinska University Hospital, 17164 Solna, Sweden.

*E-mail: siddharth.sahu@angstrom.uu.se, apurba.dev@angstrom.uu.se

S1. Fluorescence measurements

Fluorescence measurements were done to check and compare the surface coverage of the captured sEVs by the APTES-GA and the PPB-avidin functionalization methods in order to check if the enhanced sensitivity comes from the electrostatic charge contrast alone or is partially due to a difference in the capture efficiency. For this purpose, sEVs were isolated from H1975 cells, stably expressing GFP-tagged CD9 (see material and methods). The

fluorescence measurements on the GFP tagged sEVs were performed with the 100x oil immersion lens of a Zeiss inverted microscope Colibri 5, equipped with a Hamamatsu CCD Camera (Orca Flash 4). The 475 nm wavelength LED was used for exciting the GFP tagged sEVs, and their images were recorded with 2s acquisition time across an area of 133.2 µm × 133.2 µm. The capture of sEVs was done following the functionalization scheme of APTES-GA and PPB-avidin, as mentioned in the materials and methods section. The resulting images are shown in the figure S1. On an average, a total of 567 sEVs were captured by the APTES-GA surface in a microscopic area, whereas 511 sEVs were captured by the PPB-avidin coated surface. The surface coverage is hence about 10% lower in case of the PPB-avidin surface. This shows that despite the lower extent of sEVs captured by the PPB-avidin functionalization scheme, the net signal when measured by the streaming current method is still higher.

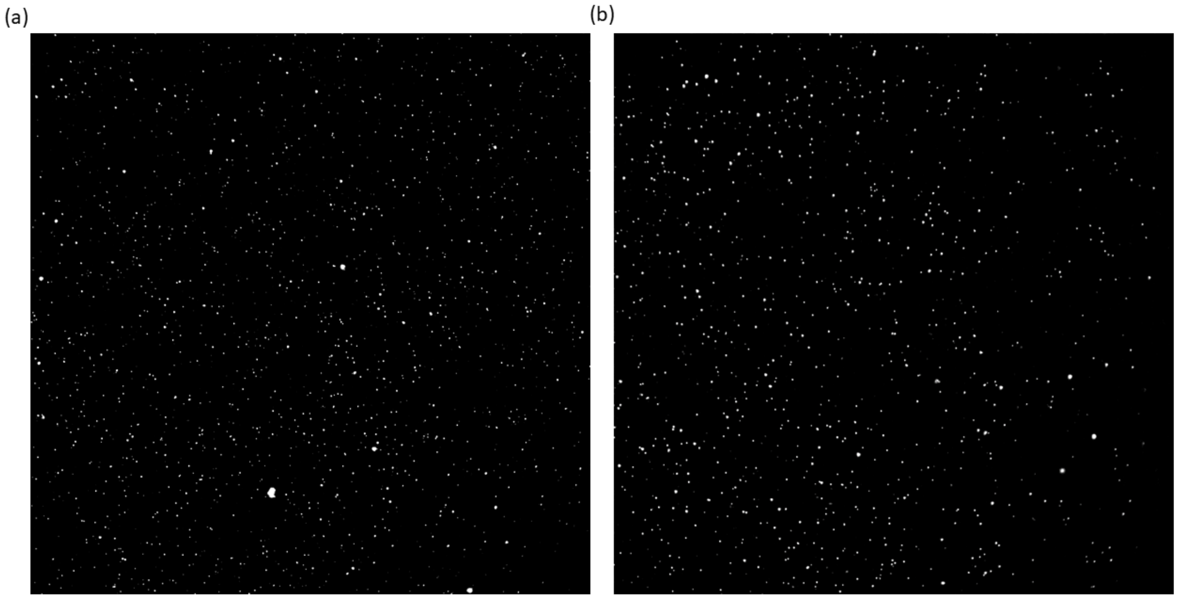

Figure S1: Fluorescence images of the sEVs from H1975 cells stably expressing GFP-CD9 captured by (a) GA-APTES and (b) PPB-avidin functionalization schemes reveal that the number of sEVs captured is about 10% less in the PPB-avidin method.

S2. Effective surface charge density

The derivation of surface charge density of a flat surface in contact with an electrolyte is possible by solving the Poisson-Boltzmann equation. The resulting solution, known as Gouy-Chapman equation, is given by[1],

$$\sigma = [8cN\epsilon_r\epsilon_0 k_B T]^{1/2}\sinh\left(\frac{e\psi_0}{2k_B T}\right)$$

… (S1)

Where c is the ion concentration, N is Avogadro's number, $\epsilon_r\epsilon_0$ is the permittivity of the medium, k_B is the Boltzmann constant, T is the temperature and ψ_0 is the surface electrostatic potential. This solution to the Poisson-Boltzmann equation however is valid only for an ideally flat surface. In reality, as the surface is not flat, this equation only provides a lower bound for the estimation of σ, rather than an accurate expression [1]. The quantity "effective charge density" can be defined as the sum of the charge density of the flat surface, as well as the surrounding ions enclosed by the slip plane. This term is related with the zeta potential, ζ_i^*, and the relation, which has been derived elsewhere, is given by,

$$\sigma_{eff} = [8cN\epsilon_r\epsilon_0 k_B T]^{1/2}\sinh\left(\frac{e\zeta_i^*}{2k_B T}\right)$$

… (S2)

Using the above equation, the value of σ_{eff} could be determined from ζ_i^*obtained experimentally for the surfaces at various stages of the functionalization, and are given in table 2. As the silica surface bore a strong negative charge in the measuring buffer to begin with, none of the functionalization methods tested here led to a stable, positively charged surface. Although the surface did become positively charged during intermediate stages of the functionalization (data not shown), it returned to a negatively charged state upon reaching

equilibrium. PPB-avidin led to the least negatively charged surface among the functionalization schemes tested, and resulted in the maximum charge contrast against the sEVs.

S3. Nanoparticle Tracking Analysis (NTA) measurements

NTA measurements were performed on the sEVs isolated from cell culture media of untreated H1975 cells, as well as those treated with erlotinib and osimertinib. The results are shown in figure S3. The diameters of the sEVs are in the range 50-300 nm. The mean diamters in case of sEVs from untreated cells, and those with erlotinib and osimertinib treatments were 201.6 ± 2.7, 182.8 ± 1.0 and 184.0 ± 0.3 nm respectively. It can be seen that the erlotinib and osimertinib treatments do not have any significant effects on the size distribution of the sEVs.

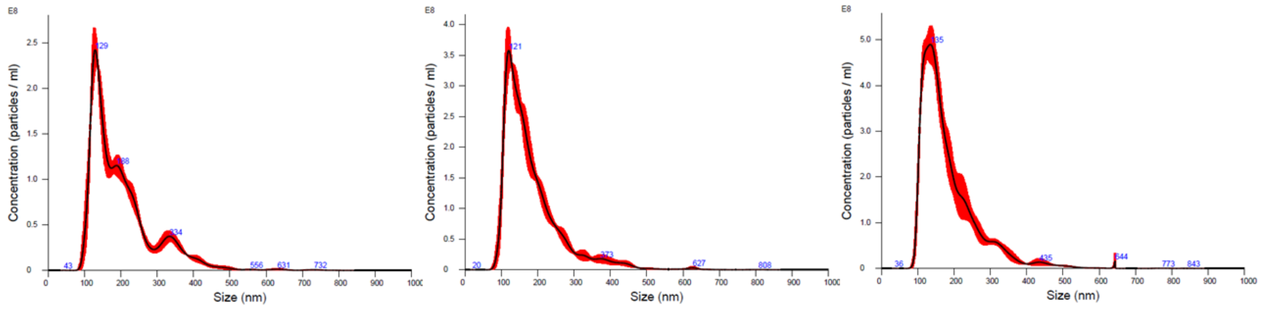

Figure S2: Nanoparticle tracking analyses of sEVs isolated from cell culture media of H1975 cells that were (a) unreated as well as those treated with (b) erlotinib and (c) osimertinib. Black lines represent the mean particle size whereas the red bars represent the standard deviation. The largest peak corresponds to a diameter of about 106 nm.

S4. EGFR-TKI Erlotinib and Osimertinib responses in H1975 cells

The response of H1975 cells to erlotinib (1µM) or osimertinib (0.1µM) was analysed at 48h post treatment when also the sEVs were harvested from cell culture media (figure S3a). As seen erlotinib at this dose had minor effect on cell morphology relative to untreated cells while osimertinib caused clear growth inhibition. The selection of these doses for erlotinib or

osimertinib were determined in preparatory experiment using MTT cell viability assay (figure S3b). As seen 1µM erlotinib didn't influenced cell viability in line with published data [2] whereas 0.1µM osimertinib reduced cell viability by approximately 50%. Western blot profiling confirmed CD9 expression in EVs prior and post EGFR-TKI treatment (figure S3c). EGFR and PD-L1 was detected in H1975 cells prior and post treatment with no major differences seen while for EVs the western blot analyses was not sensitive enough to detect the protein (data not shown).

(a)

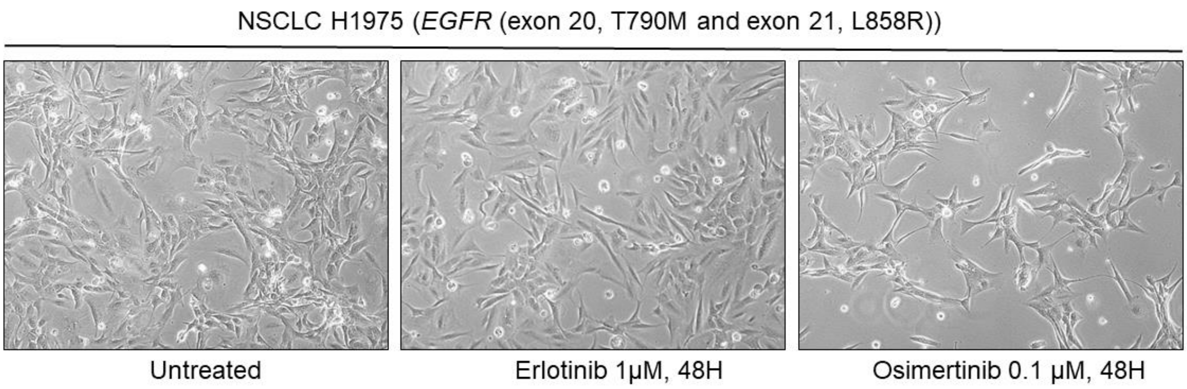

(b)

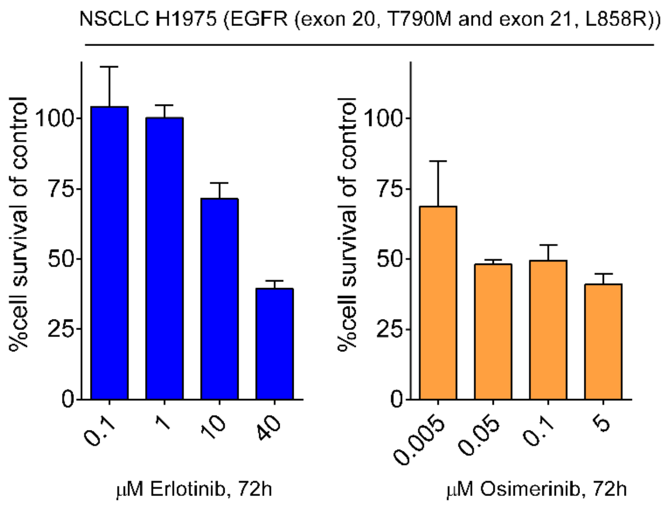

(C)

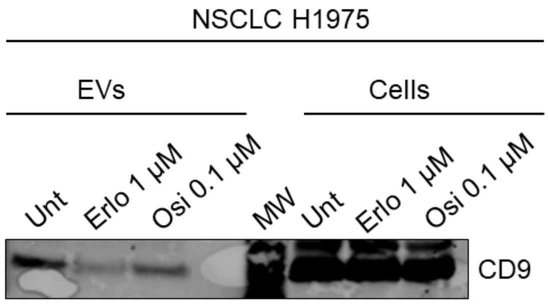

Figure S3: EGFR TKI response in NSCLC H1975 cells. (a) The morphology of the H1975 cells after EGFR-TKI erlotinib (1 µM) or osimertinib (0.1µM) for 48h and at the time point of sEVs harvesting from cell culture media was analysed using a Nikon Eclips TS100 microscope using a 10x lens. Images were further processed in Adobe Photoshop. (b) Cell viability profiling by MTT-assay at 72h. H1975 cells were treated with indicated concentrations of erlotinib or osimertinib for 72h. A DMSO-control was used for relative calculation of cell viability. (c) Western blot analyses of CD9 in EVs and H1975 cells prior and post EGFR-TKI treatment. Equal amount of EVs were loaded in the first three lanes and equal amount proteins from the cell lysates in the last three lanes.

S5. Limit of detection (LOD)

The LOD was estimated as the concentration of the target corresponding to the MDS level of the sensor. This was estimated from the calibration curve in a semi-logarithmic scale as shown in figure S4, by considering the point of intersection between the calibration plot and the MDS level (0.1 mV) of the sensor. The linear regime of the sensor response was considered for this purpose. The LOD was obtained to be 4.9×10^6 particles/mL. This is an improvement of about two orders of magnitude over the LOD obtained by us previously [3]. The LOD estimation was done using CD9 surface protein in the present study and EGFR surface protein in the previous study. As the signals in both cases are in the same order of magnitude, this does not affect our claim of improvement in LOD.

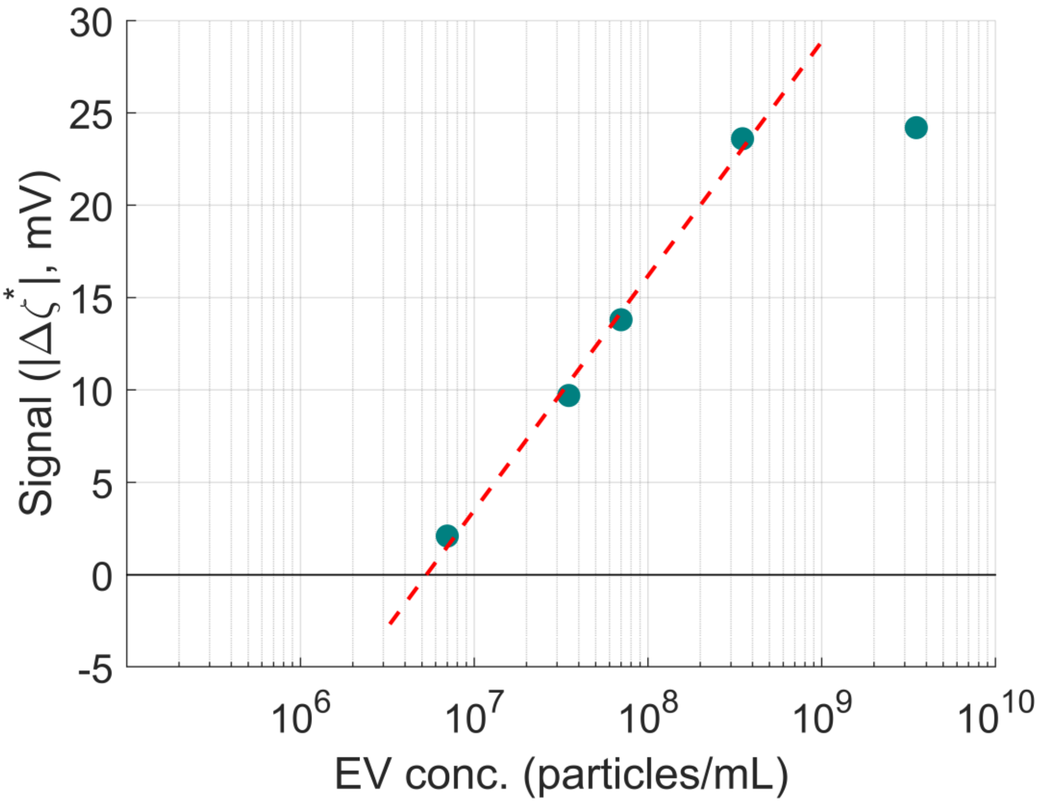

Figure S4: The limit of detection (LOD) of the sensor obtained by taking the point of intersection of the linear part of the calibration plot and the MDS level (0.1 mV) of the sensor.

S6. Simulation parameters

The Adamczyk model [4] was used for the simulations, according to equation 1. Given the fact that the Debye length is 2.3 nm in our case, and the average radius of the sEVs is much higher (~50 nm), the values of the parameters C_i and C_p lie in the saturation range according to the figure 8 of the reference [4], and were taken to be 6.5 and 10.2 respectively. Moreover, according to the results of our previous work [5] that the roughness of the surface requires a reduction in C_i, we made it 2 times smaller. The ζ_p of the EVs was assumed to be -30 mV. In reality, EVs are very heterogeneous in terms of their surface protein profiles, which can influence their ζ_p. This was ignored for simplicity.

S7. sEV isolation and characterization

In this study sEVs were isolated from cell culture media of the NSCLC cell line H1975 (ATCC® CRL-5908™, LGC Standards, Wesel, Germany). This cell line is established from a NSCLC adenocarcinoma patient and has two different mutations in *EGFR* (exon 20, T790M and exon 21, L858R) rendering it resistant to erlotinib but sensitive to osimertinib.[2,6] The cells were cultured in RPMI-1640 medium with addition of fetal bovine serum (FBS, 10%) and 2 mM L-glutamine (Gibco,Life Technologies, Stockholm, Sweden).

Prior to sEVs isolation from cell culture media the untreated cells were maintained for 48 h in RPMI media with exosome-depleted FBS (#Gibco™A2720801, Thermo Fisher Scientific, Stockholm, Sweden) and 2 mM L-glutamine to avoid contamination of FBS sEVs. For the EGFR TKI experiment the cells were plated in 175 cm^2 flasks (Sarstedt, Helsingborg, Sweden) in RPMI media with 10% FBS and 2 mM l-Glutamine. After 24 h the media was replaced to RPMI media containing 10% exosome-depleted FBS and 2 mM l-glutamine and the cells were treated with either left untreated or treated with EGFR-TKIs: 1 µM erlotinib (OSI-774) or 0.1 µM osimertinib (AZD9291) for 48 h. Both EGFR-TKIs were purchased from Selleckchem, TX, USA and diluted into a 10 mM DMSO stock with further dilution in cell culture media upon use. sEVs were isolated from 50 ml of cell culture media with two steps of centrifugation to clear out cell debris (200 RCF for 5 min followed by centrifugation of supernatant at 720 RCF for 10 min, Rotina R38 centrifuge, Hettich). Media was concentrated to about 500 µl using Amicon Ultra-15 Centrifugal Filter Unit with a MWCO of 3 kDa (#UFC900324, Merck Chemicals and Life Science AB, Solna, Sweden). sEVs were isolated by size-exclusion chromatography (SEC) on qEVoriginal columns (Izon Science, Oxford,UK) as previously described.[7] Briefly, the samples were added to the column and were eluted in 500 µL fractions by 0.22µm filtered PBS. Fractions 6-10 were pooled and concentrated to about 500 µL using an Amicon® Ultra-4 Centrifugal Filter Unit

(#UFC800324, Merck Chemicals and Life Science AB). The particle size and amount were characterized by nanoparticle tracking analysis (NTA) carried out on a NanoSight NS300 instrument (Malvern Panalytical, Malvern, UK). For these analyses the sEVs samples were diluted 1:50 in filtered PBS. The setting on the NTA was as follows: syringe pump speed: 100, camera level: 13, threshold for analysis set at 5 and time for analysis 3x60 s. The sEVs' characterization from this isolation procedure including western blot analyses of CD9 and EGFR has previously been reported.[7] sEVs were also isolated from PE fluid of two NSCLC patients with adenocarcinoma, PE002 and PE011 respectively. These two patients have an *EML4-ALK* variant 3 (a/b) alteration (PE002) and an *EGFR* exon 21, L858R mutation (PE011[7]). The PE fluid samples were obtained at Karolinska University Hospital, Stockholm, Sweden under ethical permit from the Ethics Review Authority in Sweden (https://etikprovningsmyndigheten.se), region Stockholm (EPN No. Dnr. 2016/2585-32/1, approval date 8th of March 2017) and with informed written consent from the patients. Biobank permits and Material Transfer Agreement to Uppsala University were obtained in alignment with Swedish legislation for biomaterial of patients. The isolation of sEVs from the PE fluid followed our previous isolation protocol[7] which also confirmed the expression of the sEVs markers CD9 and TSG101 in both samples as well as expression of EGFR in PE011 sEVs but not in PE002. The expression of CD9 (#13403, Cell signaling, Bionordika, Stockholm Sweden), in the untreated, erlotinib or osimertinib treated H1975 cells were analysed by western blotting. The cells were lysed using RIPA buffer (25mM Tris-HCl pH 7.4, 150mM NaCl, 1% NP40, 2mM EthyleneDiamineTetracetic Acid (EDTA), 0.1% Sodium dodecyl sulfate (SDS) containing PhosSTOP and cOmplete Mini, EDTA-free, both from Roche). The cell lysates were run on 4-12% Bis Tris gels using MES buffer (all from NuPage, Thermo Fischer Scientific). The proteins were transferred to nitrocellulose membranes (LI-COR) using transfere buffer (NuPage, Thermo Fischer Scientific) containing

10% Methanol and were blocked using Intercept blocking buffer: TBST at a 1:1 dilution. The primary antibodies were diluted in the similar buffer. The binding of primary antibodies was visualized using 800CW IRDye® Goat anti-Rabbit (926-32211) or IRDye® 680RD Donkey-anti Mouse (926-68072) on the Odyssey® Sa Infrared Imaging System (all from LI-COR).

For fluorescence experiments of sEVs, H1975 NSCLC cells were stably transfected with a CD9-GFP plasmid (Origene #RG202000). Briefly, cells at around 70% confluence were transfected using Lipofectamine 2000 (Thermo Fisher Scientific) and 0.7 ng plasmid. Plasmid uptake in the cells were selected using Geneticin (G418, Thermo Fisher Scientific). The isolation of sEVs from media from these cells were performed similarly as described for H1975 cells above.

S8. Cell morphology and cell viability analyses post EGFR-TKI treatment

The cell morphology of H1975 after 48h treatment with either erlotinib or osimertinib was analysed using a Nikon Eclips TS100 microscope using a 10x lens. The erlotinib or osimertinib induced cytotoxicity on H1975 cells were studied using 3-(4,5-dimethylthiazol-2-yl)-2,5 diphenyltetrazolium bromide (MTT) assay. Thus H1975 cells were plated at a density of 5,000 cells/well 96-well plate using cell culture media and supplements as indicated above. The next day cells were treated with indicated concentrations of erlotinib or osimertinib diluted in DMSO for 72 h. Cells treated with equal amount DMSO as used in the highest concentration of the TKIs were handled in parallel. At 72 h post drug addition, MTT solution 10 μL/well (Sigma-Aldrich, concentration 0.5 mg/mL) were added to the cells and the plate incubated at 37°C and 5% CO_2 for 2 h. By adding stop solution (10% SDS and 0.01 mol/L HCl), the formazon crystals generated were dissolved and monitored at 595 nm in the SpectraMax® I3 platform (Molecular Devices, Copenhagen, Denmark). The absorbance, which is proportional to the number of viable cells, was quantified and the relative cell

viability was calculated setting DMSO-treated cells to 100% viability. Data presented is from three biological experiments done in triplicate. Mean and SEM are shown.

S9. Preparation of biotinylated EGFR-binding antibody

The EGFR-binding antibody cetuximab (Erbitux, Merck) was biotinylated with NHS-activated biotin (EZ-Link™ Sulfo-NHS-LC-Biotin, Thermo Fisher Scientific) that reacts with primary amino groups in the antibody. 0.5 mg/ml cetuximab was incubated with 6 times molar excess of biotin for 30 minutes at room temperature. After the biotinylation reaction, the mixture was purified and buffer exchanged to PBS with Amicon Ultra Centrifugal 100k Filter (Merck). To test if the biotinylation was successful, bead capture test was performed. 2 µg of biotinylated cetuximab was mixed with 40 µl streptavidin-coated magnetic beads (Dynabeads™ M-280 Streptavidin, Thermo Fisher Scientific) and incubated for 1 h at room temperature. The beads capturing the biotinylated antibodies were mixed with 5x reducing SDS-PAGE loading buffer and incubated for 20 minutes at 95 °C. The sample then was loaded onto an SDS-PAGE gel and stained with Coomassie blue to analyse the protein content.

References

(1) Ge, Z.; Wang, Y. Estimation of Nanodiamond Surface Charge Density from Zeta Potential and Molecular Dynamics Simulations. *J. Phys. Chem. B* **2017**, *121* (15), 3394–3402. https://doi.org/10.1021/acs.jpcb.6b08589.

(2) Pao, W.; Miller, V. A.; Politi, K. A.; Riely, G. J.; Somwar, R.; Zakowski, M. F.; Kris, M. G.; Varmus, H. Acquired Resistance of Lung Adenocarcinomas to Gefitinib or Erlotinib Is Associated with a Second Mutation in the EGFR Kinase Domain. *PLoS*

Med. **2005**, *2* (3), 0225–0235. https://doi.org/10.1371/journal.pmed.0020073.

(3) Cavallaro, S.; Horak, J.; Hååg, P.; Gupta, D.; Stiller, C.; Sahu, S. S.; Görgens, A.; Gatty, H. K.; Viktorsson, K.; El-Andaloussi, S.; Lewensohn, R.; Eriksson Karlström, A.; Linnros, J.; Dev, A. Label-Free Surface Protein Profiling of Extracellular Vesicles by an Electrokinetic Sensor. *ACS Sensors* **2019**, *4* (5), 1399–1408. https://doi.org/10.1021/acssensors.9b00418.

(4) Adamczyk, Z.; Sadlej, K.; Wajnryb, E.; Nattich, M.; Ekiel-Jezewska, M. L.; Bławzdziewicz, J. Streaming Potential Studies of Colloid, Polyelectrolyte and Protein Deposition. *Adv. Colloid Interface Sci.* **2010**, *153* (1–2), 1–29. https://doi.org/10.1016/j.cis.2009.09.004.

(5) Sahu, S. S.; Stiller, C.; Cavallaro, S.; Karlström, A. E.; Linnros, J.; Dev, A. Influence of Molecular Size and Zeta Potential in Electrokinetic Biosensing. *Biosens. Bioelectron.* **2020**, *152* (December 2019). https://doi.org/10.1016/j.bios.2020.112005.

(6) Tang, Z.; Du, R.; Jiang, S.; Wu, C.; Barkauskas, D. S.; Richey, J.; Molter, J.; Lam, M.; Flask, C.; Gerson, S.; Dowlati, A.; Liu, L.; Lee, Z.; Halmos, B.; Wang, Y.; Kern, J. A.; Ma, P. C. Dual MET-EGFR Combinatorial Inhibition against T790M-EGFR-Mediated Erlotinib-Resistant Lung Cancer. *Br. J. Cancer* **2008**, *99* (6), 911–922. https://doi.org/10.1038/sj.bjc.6604559.

(7) Stiller, C.; Viktorsson, K.; Gomero, E. P.; Hååg, P.; Arapi, V.; Kaminskyy, V. O.; Kamali, C.; De Petris, L.; Ekman, S.; Lewensohn, R.; Karlström, A. E. Detection of Tumor-associated Membrane Receptors on Extracellular Vesicles from Non-small Cell Lung Cancer Patients via Immuno-pcr. *Cancers (Basel).* **2021**, *13* (4), 1–21. https://doi.org/10.3390/cancers13040922.
